# Presentations of the Acute Scrotum During the COVID-19 Pandemic: Experiences of a Non-urological Centre

**DOI:** 10.7759/cureus.28069

**Published:** 2022-08-16

**Authors:** Heather Davis, Ashim Chowdhury, Charlotte Burford, Cathy Praman, Veera Allu

**Affiliations:** 1 General and Colorectal Surgery, William Harvey Hospital, Ashford, GBR; 2 General Surgery, William Harvey Hospital, Ashford, GBR; 3 General Surgery and Anaesthesia, William Harvey Hospital, Ashford, GBR

**Keywords:** covid-19, non-urological centre, testicular exploration, testicular torsion, scrotal pain

## Abstract

Introduction: Testicular torsion is a time-sensitive surgical emergency. Assessment of the acute scrotum warrants rapid surgical review and low threshold for surgical intervention.

Materials and methods: A retrospective cohort study was undertaken for a three-month period during the first wave of the 2020 COVID-19 pandemic and the corresponding period in 2019. Data were collected for all scrotal explorations undertaken at our non-urological centre during this time.

Results: Sixteen scrotal explorations were performed from April to June 2019, one had testicular torsion (6.25%). Forty-one explorations were performed from April to June 2020, nine had testicular torsion (21.95%). The most common diagnosis was epididymitis or epididymo-orchitis in 2019 and 2020 (27% and 37.5%, respectively). Sixty-two percent of patients presented within 12 hours in 2019 compared to only 37% during the first wave of the coronavirus disease 2019 (COVID-19) pandemic.

Conclusion: This study found an increase in the number of patients presenting with acute scrotal pain triggering surgical intervention and the number of patients with testicular torsion. This is likely to reflect a decrease in patients able to access primary care assessment but may also be related to COVID-19. There was a marked delay in the presentation which has significant implications for testicular viability.

## Introduction

Testicular torsion is a surgical emergency, regarded as equivalent to life or limb-threatening conditions. Delay to treatment or misdiagnosis may cause testicular infarction and loss of affected testis [1,2], with subsequent risk of impaired fertility [3]. Testicular torsion is most commonly seen in neonates or males following puberty but has been reported in all ages [4,5]. It is caused by twisting of the spermatic cord and subsequent occlusion of blood supply. In the non-neonate, this is typically due to bell-clapper deformity, whereby the tunica vaginalis abnormally extends above the testes preventing attachment to the posterior scrotal wall. The testes are then freely mobile on the cord and can twist on the cord structures. Typical presentation is with sudden onset of severe testicular pain and one of the common causes of acute scrotum. However, the diagnosis of testicular torsion is not always simple. The annual incidence is reported at approximately 3.5 per 100,000 male children [6]. Data from the United Kingdom has demonstrated the percentage of positive scrotal explorations to be highly variable between 13% and 51% [7-9]. Common mimics or conditions to consider include torsion of hydatid of Morgagni (otherwise known as the testicular appendage) and epididymo-orchitis. However, other diagnoses such as hydrocele, idiopathic scrotal oedema, and Henoch-Schonlein purpura are also important differentials [10-12]. Salvage rates depend on the duration of surgery since the onset of symptoms and degree of twisting [13,14]. Viability is rare if symptoms persist beyond 24 hours [15]. As per guidance from the Royal College of Surgeons [16], to ensure rapid assessment and treatment of acute scrotal pain, community referrals with suspected testicular torsion should present to the nearest hospital to them, regardless of age or surgical availability. At non-urological centres, patients are seen in the emergency department by the general surgical team if there is suspicion of testicular torsion, as is the case at this district general hospital in the South-East of England. Both adult and paediatric patients are seen, however, due to clinical and anaesthetic complexity, those younger than three years old are typically referred to a tertiary centre for discussion and/or treatment. It has been reported that the coronavirus disease 2019 (COVID-19) pandemic has led to a delay in presentation for a number of serious pathologies, including acute stroke [17], complicated appendicitis [18], and myocardial infarction [19]. This could partly be attributed to the fear of attending hospital during a global pandemic and the risk of exposure to COVID-19. This study set out to determine the impact of the COVID-19 pandemic on patients presenting with suspected testicular torsion.

## Materials and methods

A retrospective cohort study was conducted comparing exploration numbers and outcomes between April and June 2020, and the corresponding period in 2019, for suspected testicular torsion. Data were taken from electronic records (TheatreMan Software and Electronic patient records). Surgical numbers and outcomes were compared between the two time periods. This study was approved by the local research and audit department (Department of Research, East Kent Hospital Trust; Registration number: SA/34/20-21) and carried out in accordance with the principles of Declaration of Helsinki [19]. No identifiable patient data was recorded and all data were kept confidential, with information governance adhered to in accordance with trust protocol.

All patients who underwent scrotal exploration at William Harvey Hospital, Ashford Kent, during the three-month period between April and June 2020 and the corresponding period in 2019 were included. Patients were excluded if testicular pain was managed conservatively (scrotal exploration was not undertaken). All scrotal exploration procedures were performed by general surgeons, in supine position under general anaesthetic. Exploration was via midline raphe incision or hemi-scrotal transverse/vertical incision as per surgeon preference. In cases of proven torsion, untwisting of the testes was done, followed by application of warm damp swabs. Where the testes were not viable, orchidectomy was undertaken. Viable testes underwent three-point fixation, with or without Jaboulay repair, as per surgeon preference. Intraoperative finding of torsion mandated exploration and fixation of contralateral testis.

## Results

**Figure 1:**
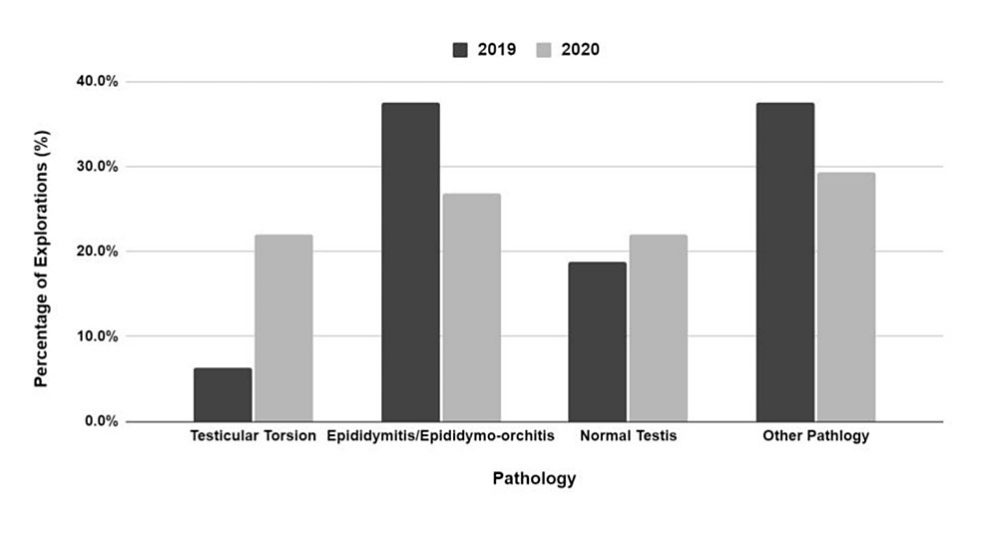
Comparison of intraoperative findings in scrotal exploration between the 2019 and 2020.

Over the three-month period within the 2020 COVID-19 pandemic, 41 scrotal explorations were undertaken, nine (22%) of which had intraoperative findings of testicular torsion and nine (22%) had intraoperative findings of normal testis (i.e., negative exploration). In the remaining 66% of cases, the most common pathological finding was epididymitis/epididymo-orchitis (n= 11, 27%; Figure 1).

By comparison, over the same three-month period in 2019, only 16 scrotal explorations were undertaken to represent a significant increase in 2020 (p <0.001, chi-squared test of independence). In the 2019 cohort, only one case (6.25%) had intraoperative findings of testicular torsion and three (19%) had intraoperative findings of normal testes. The most commonly identified pathology was epididymitis/epididymo-orchitis (n=6, 37.5%; Figure 1). The difference in rates of testicular torsion between the two cohorts was not statistically significant (6.25% vs 22%; p = 0.253, Fisher’s exact test). In 2020, the median patient age was 17.5 years (interquartile range {IQR}: 14-25) compared to 13 years old (IQR: 11-16) in 2019. This difference was statistically significant (p = 0.018, Mann-Whitney U test).

**Figure 2:**
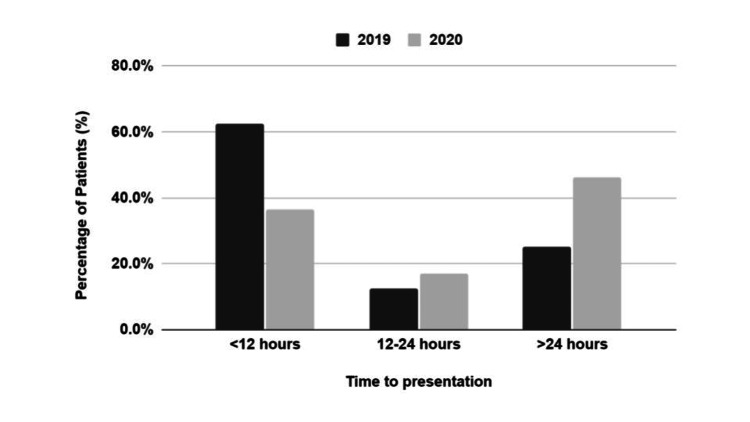
Comparison of the time to presentation in patients with acute scrotum between 2019 and 2020.

Duration of symptoms varied in both periods. In 2019, 62.5% (n=10) of patients presented within 12 hours, widely considered the period for viability, whilst 25% (n=4) of patients presented with symptoms persisting beyond a day. In 2020, 37% (n=15) of patients presented within 12 hours whilst 46% (n=19) of patients presented with symptoms persisting beyond a day. There was no statistically significant difference in the percentage of patients presenting after more than 24 hours between the two periods (p = 0.229, chi-squared test; Figure 2).

## Discussion

In this single-centre study, there was a significant increase in the number of patients presenting with an acute scrotum (16 vs. 41, p <0.001). This represents a clinically significant increase in the amount of theatre time and operating staff costs devoted to patients with acute scrotum. In a non-urological centre, this has implications for case prioritisation as these cases must be managed by the same clinical teams managing other acute general surgical pathologies, including bowel obstruction and perforation. The average age of patients presenting with acute scrotum was also significantly higher (17.5 vs. 13 years, p = 0.018) in the COVID-19 period compared to the pre-COVID period. Together this could reflect an increase in the incidence of acute scrotum, particularly in older males. Indeed, one small study of 91 patients reported an increased incidence of testicular pain in patients with COVID-19 infection although only one patient had epididymo-orchitis [20]. It is possible that the SARS-CoV-2 virus may cause testicular pain as the angiotensin-converting enzyme 2 (ACE2) is found on many testicular cell types and it has been reported as a presenting symptom in patients who are COVID-19 positive [21].

However, it is also possible that this increase is the result of alterations to the patient pathway. As a result of the constraints imposed by the global pandemic, fewer patients were able to have in-person/face-to-face reviews in the community setting. Primary care acts as the gatekeeper to secondary care and so it is reasonable to anticipate that altered working in primary care to be reflected in altered frequency of presentations to secondary care. Given the increased number of negative explorations undertaken during this period, it would indicate the exceptional triage skills of general practitioners in directing patients appropriately under normal working conditions. As general surgeons at a non-urological centre, our experience is predominantly limited to emergency presentations with an appropriate bias towards surgical exploration in the face of uncertainty.

This study also found an increase in the percentage of cases of testicular torsion and an increase in the time to presentation in the COVID-19 period, although this was not statistically significant. The increased time to presentation could be attributed to fears of attending the hospital during a global pandemic for fear of exposure to COVID-19 but also a desire not to overburden the healthcare system unless absolutely necessary. In the United Kingdom, much of the early public health messaging focused around keeping people out of hospital to reduce the burden on healthcare services. Combined these two factors may explain the delay in presentation seen in this cohort and as described earlier, a delay in presentation has been seen in other serious pathologies as well [5,20,21]. The increased time to presentation, whilst not statistically significant, is of serious clinical concern.

Whilst patient fears of contracting COVID-19 in the hospital environment may be valid, this may be at the cost of testicular viability and fertility, with lifelong implications for young patients. Moreover, testicular exploration is typically a simple procedure and most patients are able to go home the next day, limiting the opportunity for exposure.

Although clinical signs can be helpful in the assessment of suspected testicular torsion, these are not without error; for instance, the literature lacks consensus on a correct and reliable method of assessing cremasteric reflex. Nor are these signs specific to testicular torsion alone (Table 1). Furthermore, thorough clinical examination of painful testes, particularly that of distressed paediatric patients can be extremely difficult and offers limited diagnostic value.

**Table 1:** Accuracy/reliability of clinical signs in the assessment of testicular torsion.

Clinical sign	Description	Accuracy/reliability of sign
Transverse testicular position	Associated with the bell clapper deformity: testes lacks normal attachment at the tunica vaginalis	More common in testicular torsion than epididymitis (25)
Absent cremasteric reflex	L1/2 reflex: stroking inner thigh - contract cremasteric muscle: elevation of ipsilateral testes	Aged <11 years: 75% sensitivity, 83.9% specificity; aged ≥11 years: 100% sensitivity, 89% specificity; absent in 30% of normal males (26)
High riding testes	Associated with the bell clapper deformity: testes lacks normal attachment at the tunica vaginalis	Seen approximately in 50% of cases (27,28)
Prehn’s sign	Pain relief on lifting testes: suggestive of epididymitis	91.3% sensitivity, 78.3% specificity (29)
Tenderness	Epididymitis tends to present with tender epididymis whereas testicular torsion tends to present with tender testicle	65.9% sensitivity, 50% specificity (30)
Erythema	Overlying skin erythema	39.5 sensitivity, 50% specificity (30)
Blue dot sign	Venous congestion of torted hydatid of Morgagni	Found in only 10-23% of cases (25,31)

There have been multiple attempts to improve or standardise the assessment of suspected testicular torsion at this unit. Certainly, the assessment ought to include urinalysis to help identification of epididymo-orchitis and consideration of sexually transmitted infections. The most frequently used imaging is Doppler ultrasound, with sensitivity and specificity upwards of 88% and 68%, respectively. Although helpful in aiding clinical decision-making, particularly if testicular torsion is felt unlikely, the risk of delay to definitive treatment must be very carefully considered. Moreover, Doppler ultrasound is not readily available at all sites, particularly out of hours. EAU guidelines warn that Doppler ultrasound may show misleading appearances in the early phases, in partial or intermittent torsion [22]. Arterial flow may still be present in cases of testicular torsion. Consensus is for exploration in the face of diagnostic doubt.

## Conclusions

We have seen a considerable increase in the number of scrotal explorations undertaken during this time, however, the data does not reflect a statistically significant increase in the number of positive explorations. There is an increase in the number of patients presenting with suspected testicular torsion to the emergency during the period of the COVID-19 outbreak (between April and June 2020). It is of interest to note that the increase correlates with the widespread introduction of telephone consultations rather than face-to-face reviews in primary care clinics in the community and a higher proportion of the population staying at home. The data demonstrate a delay in time to presentation which may carry implications for testicular viability in cases of positive exploration. Given the possibility of future waves of the pandemic, or even future pandemics, it is imperative that public health initiatives, including education in young males, highlight the importance of presenting early with symptoms of acute scrotum.
